# Machine Learning-Based Fragility Assessment of Reinforced Concrete Buildings

**DOI:** 10.1155/2022/5504283

**Published:** 2022-08-25

**Authors:** Abdur Rasheed, Muhammad Usman, Muhammad Zain, Nadeem Iqbal

**Affiliations:** ^1^Department of Civil Engineering, MY University, Islamabad, Pakistan; ^2^School of Civil and Environmental Engineering, National University of Sciences and Technology, Sector H-12, 44000 Islamabad, Pakistan; ^3^Department of Computer Science, Abdul Wali Khan University Mardan, Mardan, Khyber Pakhtunkhwa 23200, Pakistan; ^4^Division of Computer Science, Mathematics and Science, Collins College of Professional Studies, St. John's University New York, New York City, NY 11439, USA

## Abstract

In the past, large earthquakes caused the collapse of infrastructure and killed thousands of people in Pakistan, a seismically active region. Therefore, the seismic assessment of infrastructure is a dire need that can be done using the fragility analysis. This study focuses on the fragility analysis of school buildings in Muzaffarabad district, seismic zone-4 of Pakistan. Fragility curves were developed using incremental dynamic analysis (IDA); however, the numerical analysis is computationally time-consuming and expensive. Therefore, soft computing techniques such as Artificial Neural Network (ANN) and Gene Expression Programming (GEP) were employed as alternative methods to establish the fragility curves for the prediction of seismic performance. The optimized ANN model [5-25-1] was used. The feedforward backpropagation network was considered in this study. To achieve a reliable model, 70% of the data was selected for training and 15% for validation and 15% of data was used for testing the model. Similarly, the GEP model was also employed to predict the fragility curves. The results of both ANN and GEP were compared based on the coefficient of determination, *R*^2^. The ANN model accurately predicts the global drift values with *R*^2^ equal to 0.938 compared to the GEP model having *R*^2^ equal to 0.87.

## 1. Introduction

Earthquake is one of the major natural disasters which has caused large destruction in many parts of the world in terms of economic and human loss [1, 2]. According to World Health Organization (WHO), earthquakes killed about 747,234 people and caused an economic loss of nearly 661 billion dollars worldwide from 1998 to 2017 [3]. Most of the fatalities in earthquakes are due to the collapse of buildings and infrastructures in seismic-prone countries [4]. Pakistan is also located in a highly active earthquake belt where many devastating earthquakes have jolted the region in the past. Some of the disastrous earthquakes include the 1945 Makran earthquake (Magnitude >8.0), the 1931 Mach earthquake (Magnitude = 7.3), the 1935 Quetta earthquake (Magnitude = 7.4), the 1974 Pathan earthquake (Magnitude = 6.0), and the 2005 Muzaffarabad earthquake (Magnitude = 7.6) [5–7]. In Pakistan, the poor infrastructure design and construction have caused dead consequences, especially the collapse of school buildings in the 2005 earthquake which has caused the death of 19,000 students and hugely affected the economy of the country [8]. Nearly 2/3^rd^ of the academic buildings were completely collapsed [9]. In Pakistan before the 2005 earthquake, the majority of the constructions were designed without incorporating the detailed seismic design provisions and use of nonengineered construction. Old practices were carried out in the design of buildings and bridges [10–14]. After the 2005 earthquake, the government emphasized the establishment of seismic codes which led to the Building Code of Pakistan [15, 16]. Researchers have performed analytical studies that a large magnitude earthquake can happen in Pakistan in the future [17]. Therefore, it is important to evaluate the structures against possible earthquakes.

The fragility curves, like many other techniques, can be used for the evaluation of the seismic vulnerability of buildings [18]. Fragility is the probability that the structural response (demand) due to earthquake ground motion exceeds the capacity of the structure [19]. These curves can be beneficial for retrofitting the structures and planning for future possible earthquakes in highly seismic zones such as Pakistan. Fragility curves can be established using judgemental, empirical, or analytical procedures [20, 21]. However, analytical techniques are the most commonly practiced methods using available past recorded earthquake data and structural details [22]. Researchers have established the fragility curves for 55-story core-wall RC buildings in the Philippines using two damage limit states: damage control and collapse prevention [23].

Extensive research has already been carried out on the development of fragility curves for buildings, bridges, and dams using numerical techniques [24]. However, limited literature is available to develop fragility curves using soft computing techniques. Moreover, it has also been observed that nonlinear fragility curves demand substantial analysis time and itself is a resource extensive job [19]. This article addresses the previously mentioned issues, machine learning techniques; i.e., ANNs and GEP were deployed to predict the results of conventional approaches [25, 26].

## 2. ANN and GEP Models

### 2.1. ANN Model

Artificial Neural Network (ANN) is a machine learning (ML) technique that works on the concept analogous to the human brain system. It was developed as a simplified model of brain function. There are billions of neurons (brain cells) that are interconnected in the human neural system, and these cells allow conveying signals from one neuron to the neighbouring ones [27]. It is used for solving complex problems as a powerful regression tool in various fields. The important feature of ANN is its ability to learn from experience and examples and to predict meaningful solutions. There are numerous applications of ANNs such as prediction, classification, data association, and filtering of data [28]. In the field of engineering, it has got a significant role in terms of time saving and computational cost [29, 30]. Limited research has been done in the field of structural engineering based on Artificial Intelligence (AI) techniques. However, researchers are interested to incorporate AI techniques in structural engineering due to their low computational cost and time saving. Huang and Huang have conducted a study on seismic fragility analysis of RC bridges using ANN [31]. Liu and Zhang have employed the ANN-based methodology for developing the fragility curves of steel frames [29]. Similarly, Neves et al. have assessed steel bridges based on structural health monitoring and optimization of civil engineering structures [32, 33].

The ANN architecture consists of three or more layers: an input layer, hidden layer, and output layer. Input data (theoretical, empirical, experimental, or combination of the three) is fed into the input layer on which the network learns and recognizes, and then it is transmitted to the hidden layer. The neuron has a particular weight, bias value, and an activation function. The output layer predicts the target or final results based on various algorithms [34, 35]. The neuron in the hidden layer collects the input data from the input layer and transmits it to the output layer as given in the following equation [26]:

(1) $$\begin{array}{c} h_{i}=\int \left(\sum_{i=1}^{m}w_{ij}X_{i}+b_{j}\right)\;j=1\text{,}2\ldots n\\ i=1\text{,}2\text{,}\ldots m. \end{array}$$

Here *w*_*ij*_ is the weight coefficient, *x*_*i*_ indicates the *i*^th^ input variable, *h*_*j*_ shows the output of the *j*^th^ neuron in the hidden layer, *b*_*j*_ is the bias of the *j*^th^ neuron in the hidden layer, and “*f*” represents an activation function.

There are various types of Artificial Neural architecture. The most common types of ANN are comprised of Single Layer Perceptron, Multilayer Perceptron (MLP), Radial Basis-Network (RBN), and Recurrent Neural Network (RNN), the Probabilistic Neural Network (PNN), and Cascade Correlation Neural Network [36].

One of the most commonly used algorithms is backpropagation, which can reduce the error function. Several error matrices are used for solving engineering problems, and selecting the appropriate error matrix is mandatory [37]. The predicted value minus the actual output value is known as the error function, and the mean square error (MSE) is employed for the determination of this difference as follows mathematically:

(2) $$\begin{array}{c} \mathrm{MSE}=\frac{\sum_{i=1}^{N}\left(O_{i-{\text{õ}}_{i}}\right)}{N}, \end{array}$$

where *N* shows the number of test data, *O*_*i*_ is the *i*^th^ predicted value, and *Ō* is the *i*^th^ desired target value. ANN model halts once the MSE is lower than the target value; otherwise the training continues and the values of weights and biases will keep being updated. Another important decision is the selection of an appropriate activation function while creating an ANN model. The most widely used activation functions are log-sigmoid and tan-sigmoid functions, which can be either linear or nonlinear. The nonlinear function enhances the nonlinear behaviour of the available data, so sigmoid, nonlinear function is adopted in this study as shown in the following equation [31]:

(3) $$\begin{array}{c} f\left(x\right)=\frac{1}{1+e^{-\propto x}}. \end{array}$$

Here ∝ is the sigmoid slope *a* = 1. The output data can be computed in the input layer as follows:

(4) $$\begin{array}{c} O_{i}=\int \left(\sum_{j=1}^{n}X_{j}\;Y_{j}+c_{j}\right), \end{array}$$

where *X*_*j*_ shows the weight component between the output layer and the *j*^th^ neuron in the hidden layer, while *C*_*j*_ is the bias coefficient in the output layer.

The Levenberg-Marquardt (LM), also known as the damped least squares method, is used for the computation of nonlinear least-square problems [38]. LM can mathematically be represented by the following equation [39]:

(5) $$\begin{array}{c} y_{k+1}=y_{k}-{\left[\Delta^{T}\left(y_{k}\right)\Delta \left(y_{k}\right){µ}_{y}I\right]}^{-1}\Delta^{T}\left(Y_{K}\right)\nu \left(y_{k}\right), \end{array}$$

where *y*_*k*+*1*_ = learning function, Λ(*y*_*k*_) = Jacobean matrix, and Ѵ(*y*_*k*_) = nonlinear function.

Bayesian Regularization, also known as backpropagation, is another ANN training algorithm that updates the weights and biases as per the LM optimization [40]. One-step secant (OSS) algorithm connects the quasi-Newton approach and conjugate gradient algorithms. It does not require computing the inverse matrix while calculating the new search direction. However, more computation is needed using the OSS algorithm as compared to conjugate gradient methods [41].

### 2.2. GEP Model

Genetic Programming (GP) is another machine learning technique that is an extended form of Genetic Algorithm (GA), introduced by Koza [42]. The GA was developed by Holland based on inspiration from Darwin's theory of evolution [43]. GP and GA are two different approaches based on solution representation. The GA gives linear strings or chromosomes of fixed length, while GP gives nonlinear strings of different sizes and shapes (parse trees). In 1999, Candida Ferreira introduced Genetic Programming, a technique that produces computer programs for modelling any phenomenon by mimicking the biological evolution [44].

In the GEP technique, the entities are used as linear strings having a fixed length (genome or chromosome), which are subsequently represented as nonlinear strings of different shapes and sizes (expression trees). Despite its fixed length, the GEP chromosome can encode expression trees (ETs) of varying sizes and shapes [44]. There are two major components in GEP, namely, chromosome or genome and expression tree (ETs). The chromosomes comprise a linear string of fixed length having one or more genes. Each gene itself is a string of fixed length, including arithmetic operations, fitted length parameters as a function sets, and a terminal set of constants. The genes in GEP consist of a head and a tail. The head includes symbols that show both functions and terminals while the tail comprises terminals only [45, 46].

The significance of the research is to employ the ML approach for the prediction and comparison of drift ratios of school buildings with the results obtained through the conventional approach. The optimized AI architecture and functions were used to develop the fragility curves. Similarly, the GEP model, including the expression trees, and equations were utilized for forecasting the drift values, which were then used in the development of fragility curves. The performance of ANN and GEP models was evaluated using MSE.

## 3. Methodology

This study focuses on the ANN and GEP-based fragility assessment of reinforced concrete school buildings located in seismic zone-4 of Pakistan that were designed after Muzaffarabad earthquake. Field data were collected from the school buildings through field visits and professional interviews with the concerned authorities. Field data containing structural information like the number of spans, beams, columns, the thickness of slabs, and location of stairs was collected by Ferreira from district Muzaffarabad for the existing RC school buildings and was verified with the structural drawings [44]. The databases containing building dimensions are tabulated in Table 1 [21]. The nonlinear incremental dynamic analysis was employed to determine the structural behaviour against the gradually scaled 20 past earthquake records depending upon the site history. The Peak Ground Acceleration (PGA) was selected as seismic intensity measure (IM). Three damage limit states: serviceability damage state (DS1), damage control limit state (DS2), and collapse prevention damage state (DS3), were defined. The fragility curves were plotted for each damage state using the IDA procedure in FEM software (PERFORM -3D).

**Figure 1:**
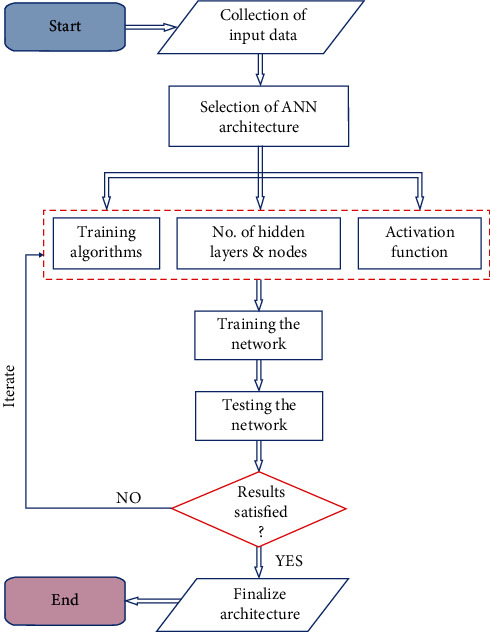
ANN flowchart with a brief description.

Furthermore, the seismic performance was predicted using the ANN model. C. Global drift (Y) is chosen as the output value in the output layer. The accuracy of the target values using the trained ANN model requires some important tasks such as the definition of a suitable ANN architecture, selection of training, testing data sets, and training and testing the network. The same architecture is trained and tested for each damage limit state. The ANN model was trained and tested in MATLAB. One of the important steps in solving the problem is choosing the optimum number of neurons and hidden layers. The percentage of error can be reduced by optimization of the number of hidden layers and the number of neurons. ANN architecture comprises a selection of training algorithms, several hidden layers, and a number of neurons and activation functions. This study utilizes a network architecture of 5 neurons in the input layer, 25 neurons in hidden layer, and one neuron in the output layer. An appropriate training algorithm is required to recognize the relationship between the input layer and output layer. In this study, three training algorithms (Levenberg-Marquardt, Bayesian Regularization, One-Step Secant) were compared, and the best one was chosen for the establishment of fragility curves. The ANN flow diagram including the collection data, selection of architecture, training and testing the network, and finally obtaining the results is shown in Figure 1.

**Figure 2:**
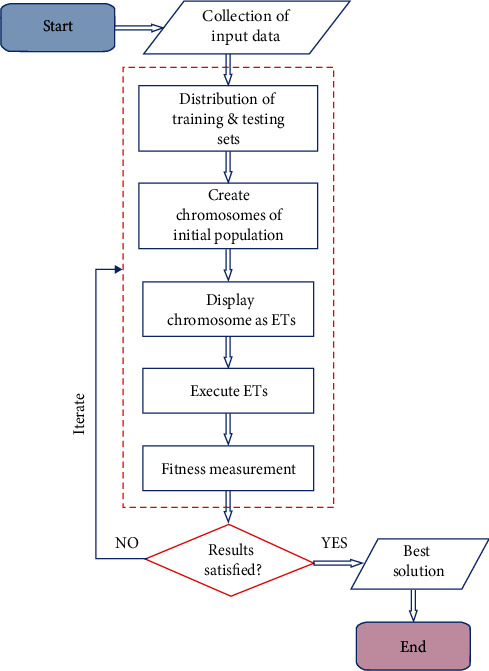
GEP flow diagram.

The drift ratios were also predicted using GEP model. The GEP model was created using three groups of fitting parameters such as general parameters, numeric constants, and genetic operators. The general parameters consist of the number of chromosomes, the number of genes, head size, and the linking functions. The numeric constants comprise constants per gene, data type, lower bound, and upper bound. The genetic operators include rate of mutation, function insertion, gene transposition, and gene recombination.  Figure 2 depicts the sequential explanation of the GEP flowchart, i.e., collection of data, distribution of data into the training and testing set, construction of chromosomes, displaying and execution of expression trees (ETs), and the measurement of fitness.

Lastly, the results of IDA, ANNs, and GEP were compared for the fragility assessment of RC buildings.

## 4. Configuration of Building Topology

The buildings considered in this study comprise 1–3 stories' buildings with different number of bays in both orthogonal directions as shown in Table 1. The size of columns and beams are different for all structures considered in this study. The height and floor area of the selected buildings are also tabulated in Table 1.

## 5. Structural Modelling

To perform the nonlinear analysis, a 3D nonlinear static model was created in CSI-Perform 3D that captures various aspects of structural behaviour such as hinge rotation and material strain. The characteristics of locally available materials were considered to achieve the actual structural behaviour as per the research done by Rafi and Nasir [47]. Grade-40 reinforcing steel was used in the analytical model as suggested by professional experts during the interviews. The nonlinear trend of the structure can be achieved if the nonlinear inelastic fibre sections are used for modelling the reinforcing steel and concrete. For this purpose, the Mander model was assigned to concrete material, and complete confinement effects were counted in the analysis [19, 48]. For steel bars, the nonbuckling steel model was used. The material and loading values are tabulated in Table 2.

## 6. Selection of Ground Motion

An important step in the development of fragility curves is the selection of appropriate past ground motion records. The target spectrum is provided by the Building Code of Pakistan (BCP) but it is not reliable. Therefore, in this study, a total of 20 different earthquake ground motions were adopted with magnitude (here 6.5–8.0), source to site distance, and the shear-wave velocity, Vs (here 175–300 m/sec), as shown in Table 3 [29]. These earthquake records were selected which resonate the site characteristics such as the fault mechanism of the Kashmir region.

The seismic intensity measure (IM) for the establishment of fragility curves is an important parameter; however, there is no clear method to decide what intensity measures to be selected. Researchers have used various IMs such as Peak Ground Velocity (PGV), Peak Ground Acceleration (PGA), and Peak Ground Displacement (PGD), and spectral response acceleration (*S*_*a*_) [49]. The intensity measures can be achieved either from earthquake records directly or by using the response spectrum of recorded earthquakes in the past which match the site history.

If the actual earthquake data is unavailable for a particular location, then a synthetic ground motion can also be developed [50]. The most frequently used intensity measure for the fragility analysis is PGA; however, it is considered that *S*_*a*_ is comparatively better [51]. Similarly, Seo et al. did the fragility analysis of curved steel bridges using PGA as an intensity measure. Tavares et al. have used PGA values for the establishment of fragility curves to evaluate the vulnerability of Canadian highway bridges [52]. The fundamental principles in the selection of accurate IMs depend on how well the hazard level of ground motion correlates the damage level of the structures. Therefore, the closeness of this correlation results in the accuracy of the fragility curves.

## 7. Structural Limit State Definition

A structure cannot fulfil its intended function once the limit state is exceeded. The structural capacity can be determined by establishing suitable limit states. Different limit states have been used by researchers in the past for the evaluation of structures. Avşar et al. employed three damage limit states: serviceability, damage control, and collapse prevention limit states for the assessment of ordinary highway bridges [49]. Researchers used four damage limit states (slight, moderate, extensive, and collapse) based on interstory drift ratio to assess steel buildings against earthquake loading [53, 54]. In this study, the global drift values are used as damage states adopted from the research conducted by Zain et al. as shown in Table 4.

## 8. Development of Fragility Curve

Fragility analysis can be defined as the probability of occurrence of damage in a structure or structural member. The probability of failure can be represented by a lognormal distribution which can be mathematically expressed as shown in the following equation [55]:

(6) $$\begin{array}{c} P_{f}\left[D\geq C\text{IM}\right]=\emptyset \left[\frac{\ln\left(IM\right)-ln\left(m\right)}{\beta }\right], \end{array}$$

where *P*_*f*_ = probability of damage occurrence, *Ø* = standard normal distribution function, *D*, *C* = earthquake demand and capacity of the structure, respectively, and IM = earthquake ground motion intensity measures (PGA, as in this study).

The terms “*m*” and “*β*” are the median value and dispersion ratio of the lognormal distribution, respectively. The lognormal distribution was used by different researchers for the prediction of structural damage due to earthquake ground motion [56].

## 9. Incremental Dynamic Analysis

IDA is one of the most reliable and effective approaches for the assessment of the structural performance of buildings subjected to earthquake ground motion. In this method, the ground motion records are scaled and then applied to the structure incrementally. The nonlinear response of the structure is captured in each step. Bayar and Bavaghar [57] adopted the IDA technique for the establishment of fragility curves for highway skewed bridges. The damage assessment of structures can be done by computing the likelihood of damage caused due to earthquake ground excitation. For this purpose, the fragility curves can be employed to quantify the probability of such damage.

## 10. Results and Discussions

### 10.1. IDA-Based Fragility Curves

**Figure 3:**
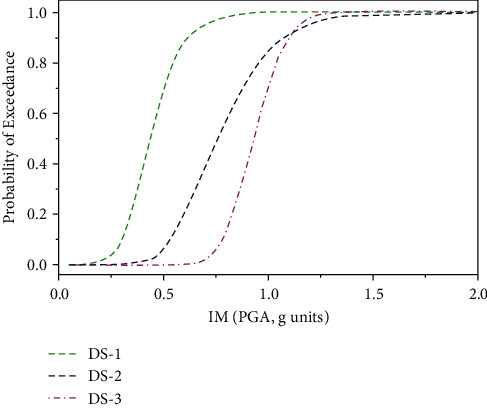
Fragility curves for three limit states using IDA approach [23].

The results of numerical analysis (NA), i.e., incremental dynamic analysis, were utilized to develop the seismic fragility curves for the building topology under consideration. The lognormal distribution function was considered for the conditional probabilities using MATLAB code. The fragility curves for three limit states: serviceability (DS-1), damage control (DS-2), and collapse prevention (DS-3), were established by plotting the IMs on axis while the probability of exceedance on the *Y*-axis is shown in Figure 3. The probability of exceedance of any limit state can be selected from the fragility curves.

### 10.2. ANN-Based Fragility Curves

The numerical analysis procedure is computationally time taking and costly; therefore, machine learning techniques might be helpful. In the current study, the fragility curves were predicted using the ANN approach.

**Figure 4:**
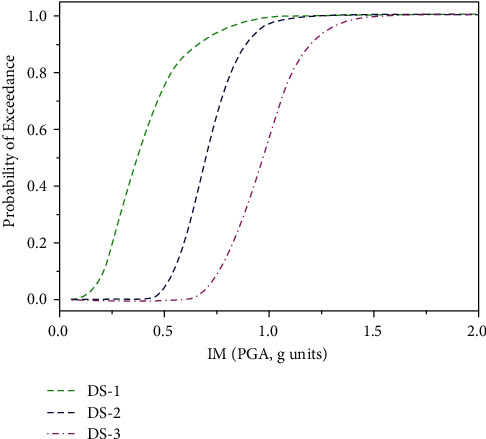
Fragility curves for three limit states using ANN approach.

The fragility curves were predicted using the ANN technique for all three damage limit states as shown in Figure 4. All three damage limit states of the ANN model show the same trends as depicted in the IDA-based approach. In order to obtain an accurate model, 70% of the data was selected for training and 15% for validation, and 15% data was used for testing the model.

Three backpropagation algorithms, LM, OSS, and BR, are compared based on their performance, i.e., mean square error (MSE) as shown in Table 5. It is vivid that the Levenberg-Marquardt (TRAINLM) is the most efficient out of all algorithms with the least MSE equal to 0.0257. Therefore, the LM training algorithm has been adopted for the ANN model.

Table 6 shows the optimized ANN architecture (5-25-1) which is selected based on hits and trials. A single hidden layer with 25 neurons is selected based on the lowest epochs and MSE values. MSE is chosen as the performance function and LM as a training function.

**Figure 5:**
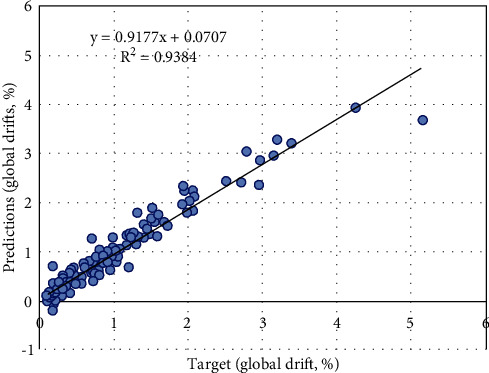
Comparison plot between target and predicted values of ANN model.

In order to predict the drift values using ANN model, a relationship is developed between the target values and the predicted values as shown in Figure 5. The performance of the model can be indicated by the correlation coefficient, *R*^2^, which is equal to 0.9384.

### 10.3. GEP-Based Fragility Curves

Selection of an appropriate GEP architecture is necessary; hence the suitable architecture was selected in this study with the optimized GEP parameters; for example, head size, number of chromosomes, number of genes, functions, and data type parameters are shown in Table 7. The number of genes and number of chromosomes are limited to 3 and 100, respectively.

**Figure 6:**
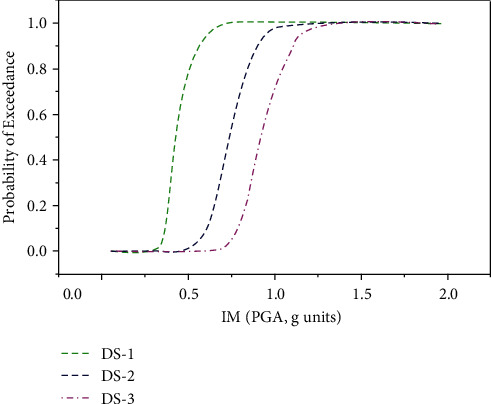
Fragility curves for three limit states using GEP model.

The fragility curves for all three limit states were plotted as shown in Figure 6, which follow almost the same trend as in the case of IDA-based fragility curves; however, compared to the ANN model, the accuracy of the GEP model is lower in terms of coefficient of correlations, *R*^2^.

**Figure 7:**
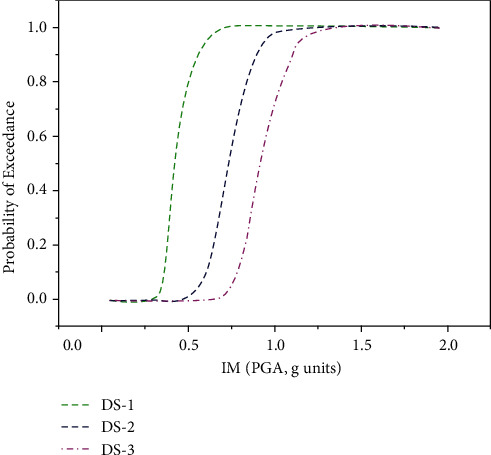
Comparison plot between target and predicted values of GEP model.

In order to predict the GEP model, the target values and predicted values of GEP model were compared as shown in Figure 7. The results obtained from the GEP model are in the acceptable range with a coefficient of correlation, *R*^2^, equal to 0.87.

**Figure 8:**
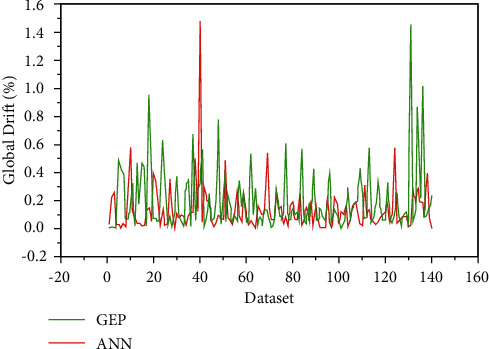
Distribution of errors of ANN and GEP models.

The error distribution between the target and predicted values is also shown in Figure 8. From the error plot, the maximum error and average error are 1.451 and 0.1953, respectively, for the ANN model. The GEP model gives the maximum error equal to 1.478 and the average error of 0.133. The maximum error in the GEP model is greater than that of the ANN model.

The global drift values (Y) can also be predicted using GEP-based empirical equation. Here equation (7) is used to forecast the global drift values. The variables A, B, and C of equation (7) can be determined using equations (8)–(10), respectively.

(7) $$\begin{array}{c} \text{Global drift values}=Y \end{array}$$

(8) $$\begin{array}{c} A=D-\left(M\times P^{2}\right)\times \left(2.504-D\right)+\frac{8.14}{N}, \end{array}$$

(9) $$\begin{array}{c} B=\frac{M\;\times \;P}{\left(N\times D\times M^{2}\right)+\left(D\times V\times P\right)}, \end{array}$$

(10) $$\begin{array}{c} C=M^{2}-\frac{\left(P+P\right)\times P}{\left(-5.87+N\right)/\left(-7.89+D\right)\;\times \left(M-N\right)}, \end{array}$$

where *Y* = global drift values, *N* = earthquake number, *D* = duration of earthquake, *M* = magnitude of earthquake, *P* = Peak Ground Acceleration (PGA), and *V* = average seismic shear-wave velocity up to a depth of 30 meters from the surface (Vs30).

**Figure 9:**
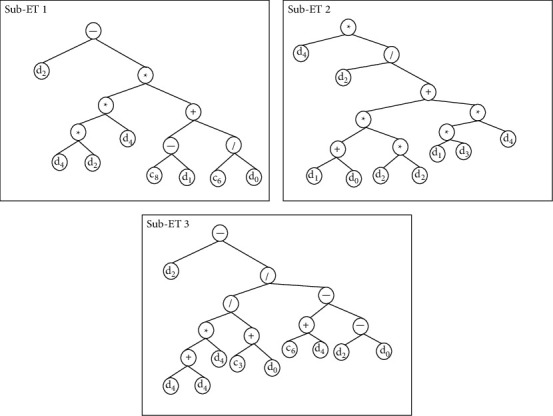
Expression trees (ETs) of gene expression programming model.

Figure 9 shows the graphical representation of the GEP model which can be decoded in the form of equations.

**Figure 10:**
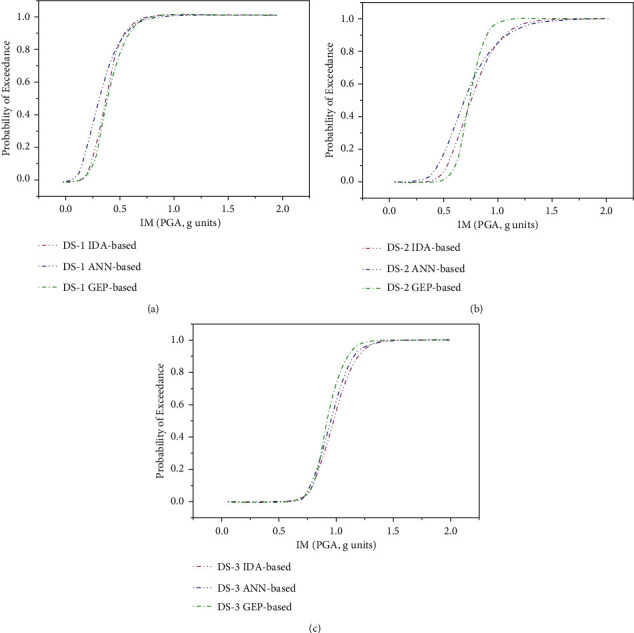
Fragility curves created by NA-based, ANN-based, and GEP-based techniques for three limit states. (a) Serviceability. (b) Damage control. (c) Collapse prevention.

The fragility curves established using incremental dynamic analysis are compared with ANN and GEP techniques corresponding to all three limit states as shown in Figure 10. In Figure 10(a), the probability of exceedance of the ANN model occurs at a lower PGA value compared to the GEP model, where the same probability of exceedance occurs at a higher PGA value. Corresponding to PGA that equals 0.4 g, the probability of exceedance is approximately 58%, 40%, and 20% using the ANN model, IDA approach, and GEP model, respectively. However, the trend reverses at PGA of nearly 0.6 g, the GEP model predicts the highest probability of exceedance followed by the IDA approach, and the lowest probability of exceedance is predicted using the ANN model. At PGA of 1.0 g and above, the fragility curves of all three models coincide, which shows the 100% probability of exceedance of the serviceability limit state.

For the damage control limit state in Figure 10(b), the ANN model follows almost the same trend as the NA model; however, the GEP model shows a high probability of exceedance at PGA greater than 0.8 g. At PGA 0.6 g, the probability of exceedance is nearly 33%, 18%, and 4%, using ANN, IDA, and GEP approaches, respectively. There is an abrupt change in the curvature of the GEP-based fragility curve at the damage limit state; however, the ANN model predicts very well at PGA 1.0 g and above.

For the collapse prevention limit state, the results of both ANN and GEP approaches forecast almost the same result as the numerical analysis-based approach as shown in Figure 10(c).

The results obtained can be used for the fragility assessment of buildings.

## 11. Conclusion

This study presents the applications of machine learning approaches such as ANN and GEP to predict the global drift values for the fragility analysis of RC school buildings in Pakistan. The results of ANN and GEP were compared with the analysis results of the incremental dynamic analysis (IDA) technique. Using soft computing techniques, the computation cost can be reduced, and the complex modelling process as in the numerical approaches can be avoided. Therefore, the machine learning techniques are useful in predicting the seismic behaviour of buildings using fragility curves.

- Out of the two ML approaches, the ANN model gives more efficient results in terms of coefficient of determination compared to the GEP model corresponding to all three limit states. The ANN model predicts the global drift values with *R*^2^ equal to 0.938 compared to the GEP model having *R*^2^ equal to 0.87.
- Both the ML models produced very similar fragility curves as were obtained using numerical modelling. Unlike the conventional numerical approaches such as incremental dynamic analysis which requires huge computational cost and is time-consuming, the ML techniques, especially the ANN approach, can be utilized to establish the fragility curves for the seismic assessment of buildings.

The performance of ML models can further be enhanced by improving the quality data and increasing the number of datasets.

## Figures and Tables

**Table 1:** Structural parameters for the selected buildings [23].

Building ID	No. of stories	No. of bays *X*-direction	No. of bays *Y*-direction	Total building height (m)	Typical floor area (m^2^)	Area of slab opening (m^2^)
BLR-1	1	6	3	3.8	328.6	—
BLR-2	1	3	3	3	101.5	—
BLR-3	1	4	2	3.8	143.1	—
BLR-4	1	4	2	3.8	146.6	—
BLR-5	1	5	2	3.6	197	—
BLR-6	1	6	3	3.6	328	—
BLR-7	1	6	3	3	306.6	—
BLR-8	1	3	2	3.8	80.8	—
BLR-9	2	4	2	7.6	145.2	9.3
BLR-10	2	4	2	7.6	125.5	9.3
BLR-11	2	11	2	6.1	344.5	21.6
BLR-12	2	2	2	6.1	66.4	6.8
BLR-13	2	5	2	6.7	192.7	16.3
BLR-14	2	8	3	6.7	253.6	23.5
BLR-15	2	3	2	6.1	58	5.7
BLR-16	2	5	2	6.1	223	7.7
BLR-17	3	4	2	11	390.2	24.8
BLR-18	3	5	2	11	209.2	15.3
BLR-19	3	12	2	9.1	377.6	21.4

**Table 2:** Material and loading values for the structural modelling.

Characteristics	Strength (MPa)	Characteristics	Load
Concrete strength, *f*_*c*_	21	Live	2.6 kN/m^2^
Reinforcing steel, *f*_*y*_	275	Infill-partitioning	4.3 kN/m

**Table 3:** Important parameters of the selected 20 past earthquake ground motions.

Sr. no	Earthquake	Year	Magnitude	PGA (g)	*R*_rup_ (kms)	Vs30 (m/sec)
1	San Fernando	1971	6.61	0.225	22.77	316.46
2	Gazli, ISSR	1976	6.80	0.864	5.47	259.59
3	Tabas, Iran	1978	7.35	0.106	28.79	324.57
4	Spitak Armenia	1988	6.77	0.20	24.0	343.53
5	Loma Prieta	1989	6.93	0.1695	24.57	239.69
6	Loma Prieta	1989	6.93	0.331	9.30	347.90
7	Northridge-01	1994	6.69	0.345	8.66	297.71
8	Northridge-01	1994	6.69	0.309	12.51	326.47
9	Chi-Chi Taiwan	1999	7.62	0.137	24.96	235.13
10	Chi-Chi Taiwan	1999	7.62	0.273	16.04	233.14
11	Chi-Chi Taiwan	1999	7.62	0.1448	25.42	297.86
12	Chi-Chi Taiwan	1999	7.62	0.165	17.11	272.67
13	Chi-Chi Taiwan	1999	7.62	0.1918	11.57	212.72
14	Kashmir earthquake	1999	7.60	0.2517	26.0	223.04
15	St. Elias, Alaska	1999	7.54	0.1759	26.45	306.37
16	Niigata, Japan	1979	6.63	0.4764	12.81	274.17
17	Montenegro, Yugoslavia	2004	7.1	0.2928	5.76	318.74
18	Chuetsu-oki, Japan	1979	6.8	0.176	29.45	334.0
19	Iwate, Japan	2007	6.9	0.2194	8.43	279.36
20	Iwate, Japan	2008	6.9	0.2057	16.67	348.98

**Table 4:** Damage limit states for consider building [23].

Limit state	Global drift ratio (%)
Serviceability (DS1)	0.35
Damage control (DS2)	0.66
Collapse prevention (DS3)	0.89

**Table 5:** Comparison of training algorithms for ANN optimization.

Sr. no.	Algorithms	Hidden layer	Performance (MCE)
1	TRAINLM	1	0.0257
2	TRAINOSS	1	0.0738
3	TRAINBR	1	0.2810

**Table 6:** Optimized ANN architecture was used for training.

Sr. no.	Parameters	Selection
1	Training function	Levenberg—Marquardt
3	Performance function	Mean square error (MSE)
4	No. of hidden layers	1
5	Number of neurons in hidden layers	25
6	Input parameters	5
7	Output parameters	1

**Table 7:** Optimized GEP parameters.

Sr. no.	Parameters	Selection
1	Head size	10
3	No. of chromosomes	100
4	No. of genes	3
5	Linking function	Multiplication
6	Fitting function	MSE
7	Constant per gene	10
8	Data type	Floating point
9	Lower bound: upper bound	−10 : 10
10	Mutation rate	0.00138
11	Inversion rate	0.00546

## Data Availability

The data used to support the findings of this study are available from the corresponding author upon request.
